# Immunological Response to Exercise in Athletes with Disabilities: A Narrative Review of the Literature

**DOI:** 10.3390/healthcare11121692

**Published:** 2023-06-09

**Authors:** Maha Sellami, Luca Puce, Nicola Luigi Bragazzi

**Affiliations:** 1 Physical Education Department, College of Education, Qatar University, Doha P.O. Box 2713, Qatar; 2 Department of Neuroscience, Rehabilitation, Ophthalmology, Genetics, Maternal and Child Health (DINOGMI), University of Genoa, 16132 Genoa, Italy; 3 Laboratory for Industrial and Applied Mathematics (LIAM), Department of Mathematics and Statistics, York University, Toronto, ON M3J 1P3, Canada

**Keywords:** immune system, disabled athletes, infection, lymphocytes, cytokines, narrative review

## Abstract

For a person with a disability, participating in sports activities and/or competitions can be a challenge for the immune system. The relationship between exercise and immunity response in disabled athletes is, indeed, extremely complex for several reasons, including (1) the chronic low-grade inflammatory and immunodepression—“secondary immune deficiency”—state imposed by the disability/impairment; (2) the impact of the disability on an array of variables, spanning from physical fitness to well-being, quality of life, sleep, and nutritional aspects, among others, which are known to mediate/modulate the effects of exercise on human health; (3) the variability of the parameters related to the exercise/physical activity (modality, frequency, intensity, duration, training versus competition, etc.); and (4) the intra- and inter-individual variability of the immunological response to exercise. In able-bodied athletes, previously published data described several exercise-induced changes affecting various immunological subsets and subpopulations, ranging from neutrophils to lymphocytes, and monocytes. Broadly, moderate intensity workout is accompanied by optimal immunity and resistance to infections such as upper respiratory tract infections (URTI) in athletes. Periods of intense training with insufficient recovery can cause a temporary state of immunosuppression, which should end with a few days of rest/recovery from exercise. Disabled athletes are relatively overlooked and understudied with respect to their able-bodied counterparts. Findings from the few studies available on paralympic and disabled athletes are here summarized and analyzed utilizing a narrative approach to review and determine the major features of the immunological and inflammatory responses to exercise in this specific population. Moreover, a few studies have reported behavioral, dietary, and training strategies that can be adopted to limit exercise-induced immunosuppression and reduce the risk of infection in people with disabilities. However, given the paucity of data and contrasting findings, future high-quality investigations on paralympic and disabled athletes are urgently needed.

## 1. Introduction

“People with disabilities” or “disabled people” are broad umbrella terms and expressions that refer to a group of individuals or a specific population that is particularly socially vulnerable and exhibits special needs. These subjects often experience societal stigma, discrimination, and marginalization, reporting as well multiple social disadvantages and either structural or perceived barriers [1,2], such as lack of accommodations and inequities/disparities in perceived quality of life and health-related outcomes, as well as in the use of/access to healthcare provisions [3,4].

According to the currently available body of scholarly research, adults living with disabilities are up to four times more likely to report their health to be fair or poor compared with their able-bodied counterparts [5]. These disparities can be due to both inherent/intrinsic and extrinsic factors, which include the type of impairment and underlying co-morbidities, and ableism, respectively [3]. The lack or scarcity of accessible information, specifically devised for people with disabilities, increases the barriers they may experience, which is dramatically impactful [3]. These factors are cascading and compound, requiring, as such, an intersectional lens to be properly addressed. Besides subjective health-related outcomes, including perceived quality of life, disabled individuals are at an increased risk of poor objective outcomes because of a “triple jeopardy”, which includes disability itself, reduced access to healthcare and rehabilitation provisions, and inadequate or ineffective policies, which are not truly disability-inclusive. For example, the efforts in terms of public health measures and the non-pharmaceutical interventions (NPIs) implemented to mitigate against the burden posed by the still ongoing Coronavirus Disease 2019 (COVID-19) pandemic have had some adverse social impacts, especially on marginalized and stigmatized communities [6].

Exercise can exert beneficial effects by enhancing the quality of life [7,8,9,10] and protecting against disorders such as communicable [11,12] and chronic-degenerative ones [13,14]. On the other hand, if acute and particularly intense, strenuous, and vigorous, exercise practiced by elite athletes can result in the insurgence of infectious diseases, muscle injuries, inflammation, or cardiovascular disorders [15,16]. The management and treatment of these accidents are hindered by a delayed diagnosis because there are no proactive policies that would enable an early, rapid, and efficient diagnosis. Continuous monitoring of the athlete is, indeed, rarely carried out. This is extremely relevant for disabled athletes and para-athletes who are at higher risk for infections [17,18] as well as chronic, non-communicable conditions [17].

There are several stressors and challenges affecting disability sports participation. Some stressors that impact athletes, including elite athletes, in general, may disproportionately and more commonly affect (elite) athletes with disabilities. Other stressors may instead be unique to (elite) disabled athletes [18]. Stressors and challenges from either or both categories include post-exercise (acute or chronic) pain, overtraining, and injury in often rather complex medical situations (with the need to distinguish sports fatigue or discomfort from sports injuries and disability). Moreover, there can also be organizational issues, including the lack of sufficient adaptive sports infrastructures or facilities, malfunctioning sports equipment, the generally high costs associated with new technologies, and logistical challenges in travel to competition sites. Other stressors are represented by challenging sleep conditions and reduced sleep quantity and/or quality in Paralympic villages or during competitions of national/international interest [19,20]. Furthermore, the pinnacle of para-sports is characterized by rapidly escalating levels of competitiveness and consequently increased training loads and demands. This pressure can also reverberate in the coaching and training environment, with some negative behaviors, such as demeaning comments, non-inclusive language, and derogatory attitudes [19]. Finally, since assignment/allocation to a given disability sports class for the competition is not fully data-driven and evidence-based, there is a risk of being misclassified or assigned to the wrong category [19]. 

Understanding the impact of these stressors and challenges on the physical and mental health of disabled and Paralympic athletes would shed light on the etiology of health-related symptoms and disorders in this specific athletic population and help devise effective symptom management and preventative strategies, accordingly [19]. In particular, these stressors can impair the immune system [21,22].

However, there is a dearth of data concerning the immunological and inflammatory response to exercise in this specific population of athletes. Therefore, to fill in this gap of knowledge, we conducted the present review of the literature.

## 2. Material and Methods

### 2.1. Search Strategy

We mined the major scholarly electronic database MEDLINE via its publicly available interface PubMed. The search string included keywords related to the immune system, disability/impairment, and the sports arena (Table 1). Truncated words (wildcard option) and Medical Subject Headings (MeSH) terms were used where appropriate. Gray literature was consulted by surfing Google Scholar. 

No time or language filters were applied. Extensive cross-referencing and hand-consultation of target journals were carried out.

### 2.2. Inclusion and Exclusion Criteria

The inclusion criteria devised according to the “Population (P)/Intervention (I)/Comparisons or Comparators (O)/Outcomes (O)” (PICOS) components were the following: studies with sufficiently trained people with disabilities, disabled athletes, and para-athletes (P); subjected to any type of training/conditioning protocol and/or vitamin supplementation or dietary strategy (I); compared across different para-sports disciplines, or with their able-bodied counterparts or stratified according to weekly training duration and frequency, internal and external training load (C); and studies reporting immunological and inflammatory response to exercise (O). The following study designs (S) were considered: quantitative investigations, either prospective or retrospective, or randomized clinical trials (RCTs). The exclusion criteria were as follows: studies recruiting only able-bodied athletes (P); rehabilitation protocols for non-athlete individuals with disabilities (I); and reporting parameters other than immunological/inflammatory ones (O). Studies designed as qualitative or lacking quantitative details, commentaries, editorials, letters to the editor, or expert opinions (S) were not deemed eligible. Review articles were not included as well but were scanned to increase the chance of obtaining all the relevant studies on the designated topic. 

### 2.3. Data Abstraction

Two independent authors (LP and NLB) designed and performed the search, screened the literature, and identified the relevant studies to be included in the present review. The agreement between the two authors was computed according to the kappa statistics and was found to be excellent. 

## 3. Results

### 3.1. Immunity and Inflammation in Disabled Individuals and Athletes: General Observations in Athletes with Spinal Lesions

In some disabilities such as spinal lesions, autonomic innervation can be altered (the so-called “autonomic dysreflexia”) with an impaired humoral and cellular immune response [23] and a systemic low-grade inflammatory state characterized by the levels of circulating inflammatory biomarkers up to 2–3 times higher than in able-bodied subjects [24]. For instance, in people with chronic spinal cord injury, the levels of certain immunoglobulins (such as IgA and IgG2) are elevated [25]. On the other hand, a case-controlled study could not find any differences in the pre- and post-vaccination ratio for IgG, IgA, and IgM response to Pneumovax 23 vaccine between injured and non-injured subjects [26]. In terms of cellular immunity, total and CD4+ T-cell frequencies were found to be increased in chronic spinal cord injury patients. CD4+ T-cells and B cells tended to shift towards memory phenotypes, in (sub-)acute and chronic spinal cord injury, respectively. Decreased immunoglobulin IgG+ and increased IgM+ B cell frequencies were found to reflect disability severity, correlating with the “American Spinal Injury Association” (ASIA) impairment scale (AIS) scores. Finally, B cell responses were comprised of an increased frequency of CD74+ cells and CD74 expression level within total B cells and B cell subsets, respectively [27]. Other studies found evidence of elevated or impaired immunological mediators [28,29].

The innate immune system is altered as well. For example, in several motor disabilities, there is a neuroinflammatory environment [30]. Interleukin 6 (IL-6), which is a single-chain glycoprotein produced and released by monocytes, endothelial cells, and adipose tissue is an activator of inflammation and a strong recruiter of immune cells after the insurgence of some injuries, including spinal cord injury [31]. This cytokine, together with other cytokines and chemokines produced by injured tissues, is released into circulation and acts as a chemoattractant for circulating monocytes which migrate to and infiltrate the injury site where they differentiate into macrophages, monocyte-derived macrophages, or MDMs [31]. It is a pro-inflammatory cytokine and, in addition, an anti-inflammatory myokine involved in an array of cellular and biochemical processes such as lipid oxidation and improvement/enhancement of insulin-stimulated glucose uptake [32]. 

C-reactive protein (CRP) is an annular, ring-shaped pentameric protein belonging to the family of pentraxins and pattern recognition receptors (PRRs). It is an acute-phase protein found in blood plasma, synthesized, and secreted by the liver, and whose circulating levels increase in response to inflammation, following IL-6 release by macrophages and T cells. It binds to lysophosphatidylcholine expressed on the surface of dead or dying cells (and some types of bacteria) to promote the activation of the complement system via C1q [33]. CRP concentrations have been found to be altered in subjects with spinal cord injuries [33,34]. Regardless of some contrasting findings reported [35], it is, however, a very common clinical observation that people with neurogenic bladder, such as those with multiple sclerosis, cerebral palsy, Parkinson’s disease, spina bifida, and spinal cord injury, are at a higher risk for indwelling urinary catheters, thus developing genitourinary infections [36,37]. Besides genitourinary issues, they are also at a higher risk for respiratory/pulmonary impairment/infection and metabolic syndrome, including overweight and obesity, dyslipidemia, diabetes, malignancies, poor wound healing, and pressure ulcers [38,39].

Specifically concerning disabled athletes, the relationship between training-related parameters, such as weekly training volume, intensity, frequency and recovery, internal and external training load, immunity, and immune system, and infection rate in the population of athletes with disabilities appears to be rather complex. In several para-sports disciplines, athletes tend to produce large training loads because of movement inefficiency and to maximize beneficial adaptions which could exert a detrimental impact on an athlete’s health by resulting in insufficient recovery [40,41], significantly impairing immunity, and thus leading to illnesses, such as infections as well as chronic-degenerative disorders.

Epidemiological surveys among disabled athletes with spinal lesions have found a higher incidence of upper respiratory tract infections (URTIs) among those with higher training loads and heavier training. For instance, Furusawa et al. [42] computed the number of URTI episodes in wheelchair marathoners during the 1 month before the race (the 18th Oita International Wheelchair Marathon) and the 2-week post-race period at 0.086 ± 0.036/week and 0.089 ± 0.040/week, respectively (versus 0.139 ± 0.046/week and 0.072 ± 0.047/week, among the controls). Even though overall, no significant differences could be detected between the before and after the race periods in marathoners, or between the two populations during each period, the number of URTIs in the 2 weeks after the race was higher in those who trained by running more than 65 km/week.

**Figure 1:**
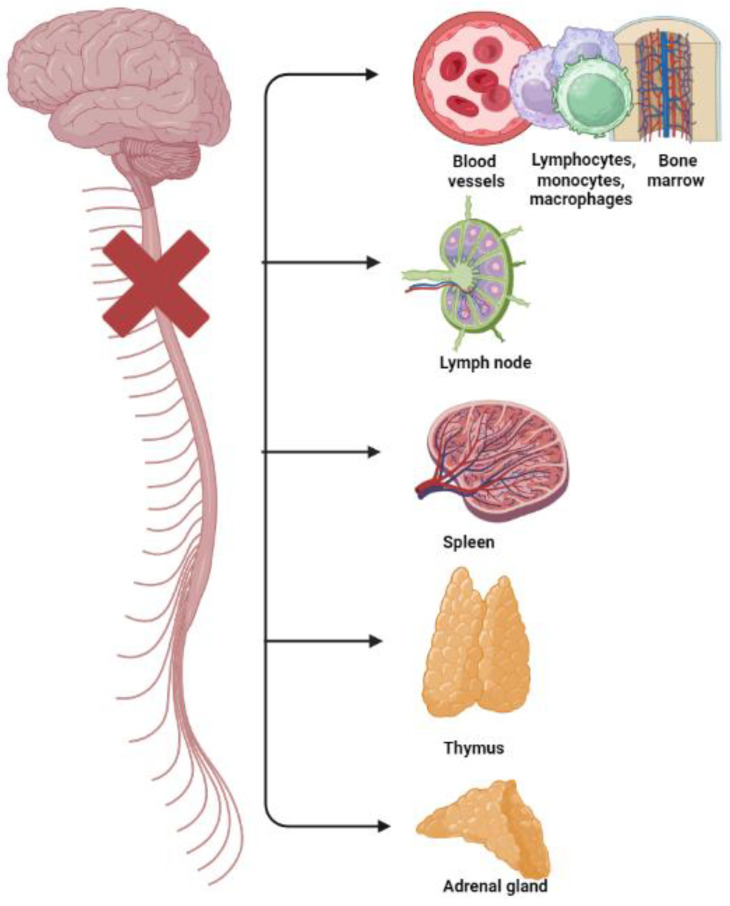
Disruption and breakdown of the outflow of signals (from the central nervous system to primary and secondary lymphoid tissues/organs—bone marrow, spleen, thymus, and lymph nodes—and to the adrenal gland, and their blood vessels; and vice versa from these tissues to the brain) causing immunological impairments in subjects with spinal cord injuries.

Disabled athletes and paralympic athletes with spinal lesions may have also high levels of hematological indexes of inflammation and platelet activation and high prevalence rates of cardiovascular disease (CVD) [17,43,44] because of autonomic dysreflexia and their poor autonomic cardiovascular control [43,44] (Figure 1).

### 3.2. Immunity and Inflammation in Disabled Individuals and Athletes: General Observations in Athletes with Intellectual Disabilities

Developmental disorders associated with intellectual disabilities [45] can show a plethora of neuro-inflammatory and/or neuro-immunological features. Of note, individuals with Down syndrome can exhibit signs and symptoms of chronic immune impairment and dysregulation, such as higher prevalence rates of immune and autoimmune/autoinflammatory disorders, which can result in hospitalization during respiratory viral infections and impose a higher burden of mortality from severe infections and complications, including pneumonia and sepsis [46,47,48]. Immune defects affect both the innate and adaptive immune systems, potentially involving every actor of human immunity from T and B cells to monocytes and neutrophils. Subjects with Down syndrome can show impaired neutrophil chemotaxis, abnormal levels of circulating cytokines, and suboptimal antibody production. Other features of the immune system may also be dysregulated such as the gamma-delta T-cell function, the inflammasome, the Toll-like receptors (TLRs), and their cascades [48].

Moreover, individuals with Down syndrome are at a higher risk of infections also due to non-immunological defects, including abnormal anatomical structures such as tracheomalacia or small ear canal, and gastro-esophageal reflux [46].

Of note, subjects with Down syndrome can have abnormal articular anatomy at the level of the upper cervical joints C1–C2, which can be foot or rockered instead of being normally cup-shaped [49]. For instance, Tassone and Duey-Holtz [50] identified other abnormalities such as hypoplasia of C1, the abnormal body structure of C2, the hypoplastic posterior arch of C1, and fusions contributing to upper cervical instabilities and potentially leading to spinal cord injuries, adding further disability.

In the next sections, we overview the effects of exercise on the mucosal humoral immune system and the non-mucosal humoral and cellular immunity.

### 3.3. Mucosal Humoral Immunity in Disabled Athletes

Leukocytes include granulocytes, lymphocytes, and monocytes. Granulocytes (neutrophils, eosinophils, and basophils) represent 60–70% of the circulating leukocytes, while lymphocytes represent 20–25% of the pool. The main types of lymphocytes are CD4+ (helper T) lymphocytes, CD8+ (cytotoxic, suppressor) T lymphocytes, B lymphocytes, and natural killer (NK) cells. B cells secrete immunoglobulins (Ig): IgA, IgG, IgM, IgD, and IgE. IgG and IgM are found mainly in plasma, while IgA is more localized in extracellular fluids within the human body, including mucous secretions. The latter type of mucosal immunity is particularly important and studied in the context of physical activity and sports. 

The adaptive humoral immune defense in mucosal secretions (saliva) and at mucosal surfaces (respiratory, gastrointestinal, and genitourinary tracts) is to a large extent mediated by secretory IgA (sIgA) antibodies, which represent the first line of defense against pathogens, especially cold-causing viruses [49]. Both saliva composition and sIgA secretion rate can be affected by parasympathetic and sympathetic nerve stimulation (the so-called “integrative autonomic-immune physiological processing” system) [51,52]. Exercise and training, by stimulating the autonomic system, can potentially lead to changes in the mucosal humoral immune compartment [52], but the impact of these alterations on health- and performance-related outcomes is still unclear.

We were able to find five studies [53,54,55,56,57] focusing on the effects of exercise on mucosal humoral immunity in disabled athletes: four [53,54,55,56] in athletes with spinal cord injuries and one [57] in subjects with Down syndrome.

Stephenson et al. [53] recruited a sample of seven elite para triathletes over 34 weeks. There was a significant negative correlation between athletes’ weekly training duration and sIgA secretion rate (*p* = 0.028) with changes in training duration accounting for 12.7% of the variance. However, no significant relationships between external or internal training load or upper respiratory tract illness (URI) and sIgA parameters could be detected. These findings contrast with previous results by Leicht et al. [54], who presented a negative correlation between training load and sIgA (*p* = 0.04) at 12 predefined time points over five months of training in a sample of fourteen elite tetraplegic athletes. No statistically significant relationship could be found between sIgA levels and subsequent upper respiratory symptoms (URS) occurrence. Finally, sIgA responses did not differ between athletes with and without URS.

In terms of sIgA responses after acute bouts of exercise, Leicht et al. [55] recruited seven highly trained wheelchair rugby athletes with tetraplegia. They carried out two separate highly strenuous sessions, lasting 23 and 41.5 min, respectively, with an aerobic or an interval focus. sIgA secretion rate and α-amylase were found to be unaffected by exercise during both sessions. On the other hand, the increases in sIgA concentration (by 67 ± 29%) 30 min after exercise during the aerobic session were accompanied by parallel decreases in saliva flow rate (by 35 ± 22%). The authors hypothesized that the disruptive impact of the disability-induced sympathetic dysfunction on sIgA secretion rate in tetraplegic athletes may be compensated by mechanisms such as reflex activity by a predominant contribution of the parasympathetic nervous system, which is intact and unaffected in tetraplegics, or by hypersensitivity of receptors.

In another study, Leicht et al. [56] recruited a sample of twenty-three wheelchair athletes, eight tetraplegics, seven paraplegics, and eight non-spinal cord-injured individuals. The athletes carried out two randomized and counterbalanced 60 min sessions on a treadmill consisting of constant load (60% peak oxygen uptake) and intermittent (80% and 40% peak oxygen uptake) exercise blocks. sIgA secretion rate and α-amylase activity were found to be increased during exercise in all groups, especially greater in tetraplegics (by 60 ± 31% versus 30 ± 35% and 11 ± 25% in paraplegics and non-spinal cord-injured subjects, respectively). 

In subjects with intellectual disabilities, Fornieles et al. [57] assessed the impact of resistance training on sIgA levels and hormone profile in 40 sedentary adults with Down syndrome, 24 of which were randomly assigned to the 12-week intervention (six stations, 3 days per week), and 16 acting as age-, gender-, and BMI-matched controls. Resistance training was found to significantly increase sIgA concentration (*p* = 0.0120; effect-size = 0.94). 

The paucity of available data and partially conflicting results concerning mucosal immunity in the population of disabled athletes warrant further research, even though studies seem to suggest a positive role of acute exercise on mucosal immunologic function in disabled athletes, regardless of the precise type of disability.

### 3.4. Cytokines and Chemokines in Disabled Athletes

Twelve studies [58,59,60,61,62,63,64,65,66,67,68,69] investigating cytokines and chemokines in disabled athletes (ten [58,59,60,61,62,63,64,65,66,67] in persons with spinal cord injuries, and two [68,69] in those with Down syndrome) could be retrieved and synthesized in the current review. 

Regarding neuromotor disabilities, Kouda et al. [58] explored the IL-6 responses to a 20 min arm crank ergometer exercise at 60% of maximum oxygen consumption in eight trained individuals with cervical spinal cord injuries (C6–C7) versus eight able-bodied trained healthy subjects. The plasma concentrations of IL-6, adrenaline, prostaglandin E_2_ (PGE_2_), and cortisol were measured before, immediately after the exercise, and one and two hours after exercise. At rest, the concentration of IL-6 was significantly higher in individuals with cervical spinal cord injuries (2.18 ± 0.44 pg/mL versus 1.02 ± 0.22 pg/mL, *p*-value < 0.05). In able-bodied subjects, the plasma IL-6 level increased significantly 1 h after exercise (1.91 ± 0.28 pg/mL, *p*-value < 0.05) and returned to the baseline level 2 h after exercise, whereas the IL-6 values were steady throughout the study in disabled individuals. The lack of exercise-related IL-6 response in individuals with cervical spinal cord injuries could be due to muscle atrophy and sympathetic nervous system dysfunction.

On the contrary, Umemoto et al. [59] investigated inflammatory response to exercise in a sample of six subjects with spinal cord injuries (T6-T10) versus seven able-bodied subjects. Both groups performed a 2 h arm crank ergometer exercise at 60%VO_2max_. Several parameters, including plasma catecholamines, IL-6, tumor necrosis factor (TNF)-α, and high-sensitivity CRP (hsCRP) were collected and measured before exercise, 60 min exercise, immediately and 2 h after the completion of the exercise. The authors determined that the arm exercise resulted in a statistically significant increase (*p*-value < 0.01) in myoglobin and plasma IL-6 levels in both groups, without any difference between the two groups. The other parameters (plasma levels of creatine kinase, lactate dehydrogenase, TNF-α, and hsCRP) did not exhibit any change throughout the study in both groups.

Kinoshita et al. [60] analyzed a sample of five wheelchair basketball players with a spinal cord injury (T7–T12) taking part in the 2009 Mei-shin League of Wheelchair Basketball Games held at Wakayama, Japan. Approximately 1 h before the player’s warm-up for the game and immediately after the game, blood samples were collected, measuring plasma IL-6, TNF-α, CRP levels, and blood cell count. Plasma IL-6 levels and the number of monocytes were found to be significantly increased after the game when compared with pre-game measurements (from 1.11 ± 0.66 pg/mL to 2.5 ± 1.29 pg/mL, and from 350 ± 246 per μL to 461 ± 266 per μL, respectively). No changes in other measurements (hematocrit, hemoglobin, red and white blood cell count) could be detected instead. Finally, the author determined a significant relationship between increased IL-6 levels and accumulated play duration (*r* = 0.94, *p*-value < 0.01), whereas no association between the monocyte count and play duration could be described. This seems to suggest that muscles, and not monocytes, can be the major source of increased IL-6 production and secretion into the blood, whilst monocytes could have been released from the vascular endothelium, as a consequence of the impact of exercise on hemodynamics. This study is limited by the absence of a control group, the low statistical power (59%), the low sample size (the recruitment of further wheelchair players would have enabled the achievement of a higher power), and the lack of serial blood measures. 

Ogawa et al. [61] studied six athletes with cervical spinal cord injuries and eight athletes with thoracic and lumbar spinal cord injuries that took part in the 30th Oita International Wheelchair Marathon Race. The authors reported stable monocyte counts in the cervical spinal cord injury group and an increase in the thoracic–lumbar spinal cord injury group, 2 h after the wheelchair half marathon. The authors also described an increase in IL-6 concentrations in both groups (but lower in the cervical spinal cord injury group) and in adrenaline levels in the spinal cord injury group, which recovered to baseline values two hours after the race. Finally, a decrease in TNF-α levels two hours after the wheelchair half marathon was noted in the cervical spinal cord injury group but not in the spinal cord injury group. Adrenaline remained stable in the cervical spinal cord injury group and was lower compared with the spinal cord injury group.

Sasaki et al. [62] found that plasma IL-6 concentrations increased by 18.4-fold and by 9.4-fold in the full and half groups immediately after the race, respectively, recovering to baseline values after 2 h, whereas plasma TNF-α and hsCRP did not change. Plasma IL-6 and hsCRP pre- but not post-race values correlated negatively with the average wheelchair speed.

Hoekstra et al. [63] investigated the relationship between autonomic function and the inflammatory response to a wheelchair half-marathon in seventeen wheelchair athletes with (*n* = 7) and without cervical spinal cord injury (*n* = 10). Catecholamine post-race levels increased only in the non-cervical spinal cord injury group (*p* = 0.036), whilst the increase in IL-6 post-race concentrations was larger in wheelchair athletes without cervical spinal cord injury (*p* = 0.040). 

Rosety-Rodriguez et al. [64] investigated a sample of 17 men with complete spinal cord injuries at or below T5, randomly allocated to the intervention (*n* = 9, a 12-week arm cranking exercise program of three sessions per week at a moderate work intensity of 50–65% of heart rate reserve) or control group (*n* = 8). Plasma levels of leptin, adiponectin, plasminogen activator inhibitor-1 (PAI-1), TNF-α, and IL-6 were gathered and measured. Plasma levels of leptin, tumor necrosis factor-alpha, and interleukin-6 were found to be significantly decreased after the completion of the training program. This study has several strengths, including its longitudinal design and random allocation (performed utilizing a concealed method).

Paulson et al. [65] analyzed a sample of twenty-six elite male wheelchair athletes (8 tetraplegics, with a level of injury at C6–C7, ten paraplegics, with a level of injury at T6-L1, and eight non-spinal-cord-injured controls). They carried out a submaximal exercise test followed by a graded exercise to exhaustion on a motorized treadmill. Blood samples were taken before, after, and 30 min after exercise and assessed for levels of IL-6, IL-10, IL-1 receptor antagonist (IL-1RA), TNF-α, epinephrine, and cortisol. Circulating IL-6 concentrations were elevated after and 30 min after exercise and increased by approximately fivefold in non-injured subjects and paraplegics (*p* = 0.003), whereas concentrations in tetraplegics did not change from baseline values. IL-10, IL-1ra, and TNF-α levels were unaffected by exercise in all groups; however, both spinal-cord-injured groups presented elevated concentrations of IL-10 compared with non-spinal-cord-injured groups (*p* = 0.001). 

Cavalcante et al. [66] assessed the effects of a wheelchair basketball-based intervention (2 h, twice a week) involving physical and tactical conditioning techniques in a sample of 48 males aged 18–55 years, 21 of which were allocated to the experimental group and 27 were allocated to the control group. The authors were able to find a significant improvement in urinary tract infections and urine culture in pre- and post-intervention antibiograms, respectively. Moreover, the intergroup comparison presented a decrease in infection caused by *Klebsiella pneumoniae*, as well as an increase in the time variability of partially activated thromboplastin, average corpuscular hemoglobin, and hemoglobin and platelets. In the experimental group, there was an increase in hemoglobin and hematocrit and a decrease in glycated hemoglobin. Regarding the intragroup comparison, there was a reduction in the levels of IL-6 before intervention and CRP after intervention.

Manns et al. [67] analyzed twenty-two men with functionally complete paraplegia (aged 39 ± 9 years, with a mean duration of injury of 17 ± 9 years and a level of injury at T2-L2). The main outcome measures were peak aerobic capacity, physical activity, and functional ability as assessed using the Physical Activity and Disability Scale and the Self-Report Functional Measure, respectively. Circulating glucose, insulin, HDL-C, triglycerides, total cholesterol, IL-6, and CRP levels were also collected and measured. The results showed that lower peak aerobic capacities correlated with lower HDL-C and lower physical activity levels, which, in turn, were associated with higher fasting glucose, lower HDL-C levels, and larger abdominal sagittal diameters. These were found to be associated with higher fasting glucose, higher fasting, and post-load insulin, lower HDL-C, higher triglycerides, and higher CRP levels.

Finally, Raguzzini et al. [68] determined that after a simulated wheelchair basketball match, growing IL-6 levels correlated with basal energy expenditure (*r* = 0.778, *p*-value < 0.05) and inversely correlated with the percentage of fat mass (*r* = −0.762, *p*-value < 0.05).

To summarize, exercise has been consistently determined to improve low-grade systemic inflammation in disabled athletes with spinal cord injuries by decreasing and increasing plasma levels of pro-inflammatory and anti-inflammatory cytokines, respectively. Slightly contrasting findings reported may be due to some methodological differences, such as the muscle mass of the participating subjects in the studies, the period of training each subject had undergone before the study, or the timing of the collection of parameters.

Regarding subjects with intellectual disabilities, Ordonez et al. [69] recruited 20 premenopausal obese young women with Down syndrome, 11 of which were randomly assigned to the intervention group (a 10-week aerobic training program, three sessions per week, consisting of a 30–40-min treadmill exercise at a work intensity of 55–65% of peak heart rate). Plasmatic levels of TNF-α, IL-6, hsCRP, and fibrinogen were assessed. Plasmatic levels of TNF-α (11.7 ± 1.6 versus 9.2 ± 1.3 pg/mL, *p* = 0.022), IL-6 (8.2 ± 1.1 versus 6.1 ± 0.9 pg/mL, *p* = 0.014) and hsCRP (0.62 ± 0.11 versus 0.53 ± 0.09 mg/dl, *p* = 0.009) significantly decreased in the intervention group. 

Rosety-Rodriguez et al. [70] recruited a sample of 40 young male adults with Down Syndrome, 24 of whom were randomly allocated to the intervention group (a 12-week program of resistance circuit training with six stations, 3 days per week). Plasma levels of leptin, adiponectin, CRP, and TNF-α were collected and assessed. They were found to be significantly decreased after the completion of the training program.

To summarize, a 10/12-week training program is effective in reducing pro-inflammatory cytokines and acute phase proteins in women and men with Down syndrome.

### 3.5. Neutrophils in Disabled Athletes

Neutrophil granulocytes play a major role in the immune system, representing the “first line of defense” against invading pathogens. Disabled people may have impaired neutrophil function in terms of altered phagocytosis and microorganism clearance. For instance, neutrophils play a key role in the pathogenesis of spinal cord injury, even if the precise cellular and molecular mechanisms underlying spinal lesions must be elucidated yet [71]. Neutrophil chemotaxis, as previously mentioned, is impaired in individuals with Down syndrome [72]. There are a few studies investigating neutrophils in disabled athletes. Most importantly, the measurement of neutrophils in response to exercise and recovery time was not yet elucidated in the existing few studies. In able-bodied athletes, moderate exercise has been shown to increase defense functions while extreme exercise decreases some without affecting others. The absolute number of neutrophils increases during and after an acute exercise [16].

For example, Levada-Pires et al. [73] recruited a sample of 10 male wheelchair basketballers aged 33.8 ± 2.7 years with a spinal cord injury (at T1-L3). Neutrophil levels and function were assessed on rest and 60 min after the basketball match. Phagocytosis capacity was found to be decreased by 75%, whereas the percentage of cells with integral plasma membrane was reduced by 17%. A fivefold increase in the proportion of cells with mitochondrial membrane polarization was reported. DNA fragmentation and phosphatidylserine externalization could not be observed. Finally, the authors described an increase in neutral lipid accumulation of 30% and a twofold increase in ROS production. The authors concluded that sport, in this specific case basketball play, can induce a significant decrease in neutrophil function (in terms of phagocytosis capacity and production of ROS) and an increase in neutrophil death (by necrosis) in wheelchair athletes. This study warrants the need for specific training and conditioning strategies for basketball players in wheelchairs and other disabled athletes to enhance athletic performance and improve disabled athletes’ health.

Of note, no article could be found exploring the effect of exercise on neutrophil function in persons with Down syndrome.

### 3.6. Natural Killer Cells in Disabled Athletes

Natural killer (NK) cells are a major class of cytotoxic lymphocytes and, more generally speaking, innate lymphoid cells (ILCs). They represent from 5% to 20% of all circulating lymphocytes in humans. 

NK cells are lymphocytes historically called “natural killer cells” because of their ability to spontaneously lyse tumors or infected cells in the absence of prior specific immunization. This property distinguishes them from CD8+ T cells (cytotoxic). NK cells are one of the components of so-called “innate” immunity. NK cytotoxicity is exerted by different mechanisms, generally such as those employed by CD8+ T cells, such as perforin-dependent cytotoxicity. Following recognition of the target cell, the NK cell degranulates: it releases at the synapse the contents of its cytoplasmic granules, especially perforin which forms pores in the membrane of the target cell [16]. Concerning NK in disabilities such as spinal cord injury, the amount of NK cells can be altered, and their cytotoxic activity (NKCA) can be found to be compromised [74]. 

A few studies [75,76,77,78,79] investigated NK cells in disabled athletes or persons with disabilities taking part in regular exercise programs. A study by Furusawa et al. [75] found that competitive wheelchair racers with a T5–L1 spinal cord injury exhibited decreased peripheral NK cells and NKCA immediately after a wheelchair full marathon. However, these parameters recovered to baseline values after just one night of rest. Different findings were reported by Furusawa et al. [76] who found a short-term increase in NKCA among disabled recreational athletes with a T7–L1 spinal cord injury after a wheelchair half marathon. The mechanism underlying such an increase was found to be adrenaline-independent [77].

Ueta et al. [78] assessed NKCA response to a 2 h arm crank ergometer exercise at 60% of maximum oxygen consumption (VO_2max_) in seven subjects with a T11–L4 spinal cord injury and six able-bodied persons. Two different NKCA response patterns could be identified, with NKCA in able-bodied subjects increasing at 60 min of exercise and immediately after the exercise, staying elevated up to 2 h after exercise, decreasing in the spinal cord injury group immediately after exercise and recovering to baseline values 2 h after exercise. Plasma adrenaline in both groups increased significantly immediately after exercise and returned to baseline level 2 h after exercise. Plasma cortisol levels were stable. In the spinal cord injury group, PGE_2_ levels significantly increased immediately 2 h after exercise and then normalized, while they were stable in able-bodied athletes.

However, Nowak et al. [79] reported an increase in NK counts in a sample of paralympic rowers who took part in the preparatory stages before the Paralympic Games in Rio, 2016. These conflicting results concerning NK immunity in the population of disabled athletes warrant further research.

### 3.7. Lymphocytes in Disabled Athletes

The amount of lymphocytes increases during exercise and decreases after long-term exercise. Studies of lymphocyte subpopulations all show a decrease in the different lymphocyte subpopulations, whether these are TCD4+, TCD8+, BCD19+, NK CD56+ (NCAM), or NK CD16+. During exercise, the CD4+/CD8+ ratio decreases due to the greater increase in TCD8+. The number of NK CD94+ or NK CD94− increases in absolute number despite a decrease in CD94. Memory T cells CD45 RO+ are preferentially recruited, and true naïve T CD45 RO-CD62L (L-selectine) increases during exercise. The study of T-cell telomeres suggests that exercise allows redistribution of activated T lymphocytes and not a repopulation from newly produced cells. While it was well described in the literature, the biphasic model of leukocytosis–lymphopenia during and after exercise and their activity during exercise is not yet explained nor described in all paralympic categories [16]. 

Ten studies [17,58,75,76,77,78,79,80,81,82] assessed lymphocyte counts in disabled athletes. Most studies could not find significant changes in lymphocyte counts before and after a sports event, except the studies by Ueta et al. [78], which reported a 1.9-fold increase (from 1.67 ± 0.6 × 10^9^/L to 3.07 ± 1.8 × 10^9^/L) and by Klokker et al. [81], in which the extent of the change was not specified. Other changes in lymphocyte counts were reported by Nowak et al. [79] in a sample of paralympic rowers. The authors found a decrease in the counts of total T lymphocytes. Moreover, shifts in T lymphocyte subsets were described with higher percentages of suppressor/cytotoxic and lower percentages of helper/inducer T lymphocyte subsets. No changes in B lymphocytes could be reported. Noteworthily, Yamanaka et al. [82] could not find any changes in total lymphocyte counts for disabled athletes with a spinal cord injury (C6–C7), but, on the contrary, computed a 1.4-fold increase for their able-bodied counterparts. Similarly, Kouda et al. [58] observed no changes in disabled athletes and reported a 1.3-fold increase in able-bodied athletes.

Finally, Bernardi et al. [17] investigated hematological indexes of inflammation and platelet activation in paralympic athletes with spinal cord injury (*n* = 25, 13 with and 12 with low spinal lesions), lower (*n* = 15) or upper (*n* = 10) limb amputation competing in power, intermittent (mixed metabolism), and endurance sports during the London 2012 and Sochi 2014 Paralympics. Inflammation scores did not differ among the groups; however, subjects with a high spinal lesion exhibited lower lymphocyte counts compared with para-athletes with lower limb amputation and higher indexes of platelet activation (mean platelet volume, platelet distribution width, ratio between mean platelet volume and platelet, ratio between mean platelet volume and lymphocyte, and ratio between platelet distribution width and lymphocyte). These data seem to suggest that para-athletes with lower limb amputation may have a higher cardiometabolic risk, whilst those with high spinal injuries may have a higher platelet-derived cardiovascular risk. 

### 3.8. The Impact of Particular Training Strategies on the Immune System in Disabled Athletes

Humans can adapt to a variety of environments. Exposure to hypoxic conditions can induce the expression of hypoxia-inducible factors (HIFs), which act as major regulators of several cellular and molecular pathways involved in processes such as cell proliferation, stem cell function, glycolysis, erythropoiesis, and angiogenesis, as well as immune regulation. More specifically, HIFs contain sequences at the level of promoter regions of genes important to immune regulation. From a clinical standpoint, hypobaric chambers can control chronic inflammation in several inflammatory conditions, including autoimmune/autoinflammatory diseases and systemic inflammation in HIV-infected subjects on antiretroviral treatment (ART). Induction of hypoxia is an effective immunotolerogenic inducer in subjects with HIV infection [83].

However, in the absence of adequate acclimatization protocols, exposure to high altitudes can result in potentially life-threatening issues, such as gastrointestinal problems including gastrointestinal tract bleeding and inflammation. In an animal model study by Khanna et al. [84], in which Sprague–Dawley rats were exposed to 7620 m of hypobaric hypoxia in an animal decompression chamber, intestinal mucosal damage could be described in terms of increased mucosal permeability and disruption of intestinal villi, as well as upregulation of sIgA and proinflammatory cytokines (IL-17) and markers including TLR type 4 (TLR-4) and inducible nitric oxide synthase (iNOS). NK cell and dendritic cell populations were found to be increased, whilst the number of naive T cells was significantly decreased in Peyer’s patches. To summarize, hypobaric hypoxia may activate the gastrointestinal–immune axis and dysregulate Th_17_ cells and proinflammatory molecules. IL-17-producing CD4+ T helper cells (Th_17_) require, indeed, to differentiate stimulation of the aryl hydrocarbon receptor (AhR) pathway, with a key role played by HIF-1-alpha (HIF-1α).

Specifically concerning disabled athletes, Park et al. [85] evaluated the effects of a 2-week exercise training program in hypobaric hypoxic conditions (week 1: simulated altitude 2000 m; 596 mmHg; week 2: simulated altitude 3000 m; 526 mmHg) created by the environmental control chambers on exercise performance (continuous aerobic exercise and anaerobic interval exercise) and immune function in six Korean national cycling athletes with disabilities. The exercise training frequency was 60 min (5 days per week). The authors determined that the number of leukocytes and NK cells significantly decreased, whilst the number of eosinophils, B, and T cells significantly increased. 

### 3.9. The Impact of Particular Dietary Strategies on the Immune System in Disabled Athletes

We were able to find three studies [86,87,88] addressing the impact of particular dietary strategies on the immune system and the inflammatory state in disabled athletes.

Marques et al. [86] investigated the effects of docosahexaenoic acid (DHA)-rich fish oil (FO) supplementation (3 g/day for 30 days) on neutrophil function in a sample of eight male wheelchair basketball players before and after acute exercise. In the absence of DHA-rich FO supplementation, acute exercise was found to lead to the loss of membrane integrity, ROS production, and high mitochondrial membrane potential in neutrophils, and reduced the phagocytic capacity and IL-6 production by the neutrophils, confirming the results of the previous study by Levada-Pires et al. [73]. These effects could be counteracted by FO supplementation. An et al. [84] conducted a double-blind, randomized, crossover study and recruited ten wheelchair basketball players (aged 34.5 ± 8.9 years; lean body mass of 34.3 ± 10.0 kg) who had spinal cord injury and had undergone amputation leucine-enriched essential amino acid (LEAA) supplementation. Of these ten players, nine participated in the final test, receiving LEAA supplements (three times 4.0 g/day), or a placebo. Parameters related to muscular fatigue and inflammatory response were measured before the intense exercise and 4 days after recovery. LEAA supplementation significantly inhibited circulating IL-6 levels (*p*-value < 0.05), without affecting TNF-α and creatinine kinase concentrations. Finally, Al-Rubaye et al. [88] performed a cross-sectional study recruiting 100 disabled athletes with hemodialysis and hemophilia. The overall dietary inflammatory index (DII) score was −2.83, indicating anti-inflammatory dietary uptake. However, no statistically significant association could be found between the DII of the participants and the components of their body composition, such as body mass index (BMI), waist circumference, waist–hip ratio, fat mass percentage, and fat-free mass percentage.

### 3.10. The Impact of Particular Pharmacological Strategies on the Immune System in Disabled Athletes

We were able to identify three studies [89,90,91] covering the impact of specific pharmacological strategies on the immune system and inflammatory state in disabled athletes. 

Aidar et al. [89] conducted a randomized, placebo-controlled trial and recruited 10 Paralympic Powerlifting athletes at the national level, aged 27.13 ± 5.57 years. They underwent a warm-up and a 5 × 5 at 80–90% of 1 RM, ingesting ibuprofen 15 min before and 5 h after training. Ibuprofen ingestion resulted in greater peak torque values (*p* = 0.04, at 24 h) and lower fatigue index (*p* = 0.01, at 24 h), whereas there was no impact on oxidative stress markers. Blood indicators, including leukocytes, with the use of ibuprofen were higher than with a placebo (*p* < 0.001). In another work, Aidar et al. [90] conducted a randomized, placebo-controlled trial and recruited a sample of 20 Paralympic powerlifting athletes (10 at the national level aged 32.50 ± 3 years, and 10 at the regional level aged 30.75 ± 5.32 years). Athletes underwent a warm-up and a 5 × 5 at 80% of 1 RM, with half of the subjects ingesting ibuprofen 15 min before the commencement of the training. Ibuprofen ingestion resulted in greater peak torque values (*p* = 0.007) and lower fatigue index (*p* = 0.002) in the national level group. Leukocytes, with the use of ibuprofen in the national-level group, were greater in concentration than in the regional-level group (*p* = 0.001). Similarly, neutrophils in the national-level group treated with ibuprofen were greater in concentration than in the regional-level group treated with ibuprofen and placebo (*p* = 0.025). Lymphocytes in the national-level group treated with ibuprofen were lower in concentration than in the regional-level group treated with ibuprofen and placebo (*p* = 0.001). Monocytes in the national-level group with ibuprofen and placebo were lower in concentration than in the regional-level group treated with ibuprofen (*p* = 0.049). Hemoglobin, hematocrit, and erythrocyte values were higher in concentration at the national level in athletes treated with ibuprofen and placebo than at the regional level in those treated with ibuprofen and placebo (*p*-value < 0.05). Ammonia levels were higher in concentration in the national-level group treated with ibuprofen (*p* = 0.007) and placebo (*p* = 0.038) compared to the regional-level group treated with ibuprofen and placebo, respectively. Fraga et al. [91] recruited eight Paralympic powerlifting athletes (aged 27.0 ± 5.3 years) competing at the national level who underwent a warm-up and a 5 × 5 at 85–90% of 1 RM. Ingestion of ibuprofen or placebo occurred 15 min before and 5 h after training. Maximal isometric force only decreased in the placebo condition, with a significant increase between 24 and 48 h in the ibuprofen condition, whilst the post-exercise rate of force development decreased significantly for both conditions. Muscle temperature decreased significantly at 48 h after exercise in the placebo condition, while deltoid muscle temperature 48 h after exercise was higher in the ibuprofen condition. Finally, creatine kinase was higher in concentration with the placebo than with ibuprofen 48 h after exercise, whilst alanine aminotransferase was lower in concentration 24 h after the training with ibuprofen. Immediately after training, aspartate aminotransferase increased with a placebo, while with ibuprofen it increased after 24 h. 

## 4. Discussion

There is a limited number of studies on the immunological response to physical activity/exercise in people with disabilities on a range of selected immunological parameters (Table 2). 

### 4.1. Mucosal Humoral Immunity in Disabled Athletes

In able-bodied athletes, several important acquired (specific) immune functions including antigen presentation by monocytes/macrophages and derived cells, immunoglobulin production by B cells, T-cell cytokine production, and proliferation (e.g., interferon-gamma), are reduced after prolonged exercise. Mucosal immune protection may also be compromised. For example, sIgA response to intense and strenuous exercise is variable, even though it is generally accepted that very prolonged exercise sessions such as triathlon may result in lower sIgA. Trying to explain these variations, several scientists claim the role of stress biological amines and hormones (including catecholamines and cortisol) together with the disruption of pro-/anti-inflammatory cytokine balance, especially in cellular environments characterized by higher IL-6 secretion.

However, for athletes with disabilities, contradictory findings could be reported. One study [53] found no significant relationships between external or internal training load or URI and sIgA parameters. These results are in contrast with other studies [54,55], which presented a negative correlation between training load and sIgA in a sample of elite tetraplegic athletes. On the other hand, no statistically significant relationship could be found between sIgA levels and subsequent URS occurrence. Finally, sIgA responses did not differ between athletes with and without URS. In addition, it is important to mention here that all samples were taken before and after the acute exercise, and it resulted in increased sIgA during recovery in tetraplegic athletes, contrary to previous studies with able-bodied athletes.

The dearth of currently available data and the reporting of partially conflicting results concerning mucosal immunity in the population of disabled athletes (see also studies [56,57]) warrant further research. In this context, it is important to highlight the role of exercise duration and intensity on humoral immunity and consider these parameters as external factors playing a key role in the modulation and fine-tuning of the adaptation to training and exercise when it comes to exploring humoral immunity responses in disabled individuals. This should be investigated together with other relevant variables, such as the concentrations of cortisol and other molecules known to influence (or confound) the expression of sIgA. Moreover, a more diverse representation of disabilities would be appreciated in that virtually all available studies focus on spinal cord injury, as such overlooking other kinds of impairment and other disabilities.

### 4.2. Neutrophils in Disabled Athletes

Based on the currently available body of scholarly data on able-bodied athletes, it appears that exercises of low to moderate intensity (60% VO_2max_) and of medium duration (approximately 60 min) exert less stress on the immune system than prolonged sessions (over 90 min) of intense effort (greater than 75% VO_2max_). Moderate-intensity exercise results in a reduced response to stress hormones, which is associated with a more favorable immune response. The increase in the number of polymorphonuclear neutrophils (innate immune cells) and the improvement in their functionality, as well as the increase in the number of monocytes and certain lymphocytes (NK cells), following moderate exercise plead in favor of an improvement of the immune defenses in response to moderate activity. Neutrophils represent about 60% of leukocytes and participate in the immune response. Their function is to migrate to infected sites where they bind, ingest, and kill pathogens. Neutrophils play an important role as mediators of inflammation. On the one hand, neutrophils release various substances that contribute to the inflammatory reaction and the recruitment of other immune system cells to the site of infection. On the other hand, recent studies have shown that neutrophils are not only involved in promoting this response, but also take part in the resolution and repair of damaged tissues.

During an infection or in the event of tissue damage such as spinal cord injuries, neutrophils communicate and interact with various cells of the immune system (macrophages, NK cells, T lymphocytes, and B lymphocytes) by “orchestrating” the evolution of immune responses, both innate and adaptive. Neutrophils activate pro-inflammatory responses eliciting macrophages that phagocyte debris and clean the damaged tissue in spinal cord injury. In addition, neutrophils can play a key role in different conditions and biological/physiological events, such as the responses and adaptations to physical exercise in individuals with disabilities.

Of interest, in spinal cord injury, Levada-Pires et al. [73] reported decreased phagocytic activity in response to intense physical activity with increased ROS production. Similarly, Marques et al. [86] reported an increase in ROS but also lower phagocytic capacity and IL-6 production. Regulation of neutrophil production and pro-anti-inflammatory status in spinal cord injury remains unclear and based on very few studies, while it is certain that neutrophils perform different important immune functions. Their inadequate response or dysregulation can also lead to the development of different disease states, including immunological impairments and chronic inflammation.

### 4.3. Lymphocytes in Disabled Athletes

In able-bodied athletes, the practice of exercises/physical activities of any intensity can produce pro-inflammatory cytokines, even though higher levels of inflammatory mediators are generally reported after intense and prolonged exercise. This stimulates a complementary anti-inflammatory response, the homeostasis of which can help clear infections and reduce inflammation, resulting in beneficial health effects. Studies found, indeed, that both moderate and intense intensity exercise may release pro-inflammatory cytokines with the subsequent promotion of anti-inflammatory molecule production. Anti-inflammatory mediators are then released to combat the pro-inflammatory response and restore immunological balance [92].

In able-bodied athletes, during exercise, increases in the number of lymphocytes have been reported in various lymphocyte subsets/subpopulations, including CD4+ or CD8+ T lymphocytes as well as CD19+ B lymphocytes and CD16+ or CD56+ NK lymphocytes. The most increased populations are NK cells and CD8+ lymphocytes, inducing a decrease in the CD4+/CD8+ ratio that can reach values in the range of 30–60% [93]. An increase in NK cells can lead to a decrease in T cells. Concerning disabled athletes, conflicting results were found in the studies conducted on the spinal-cord-injured (paraplegics or tetraplegics) in the modulation of the response and adaption to exercise and overviewed in the present review.

Most of these studies measured the lymphocyte count before, immediately after the exercise/competition, and during recovery. Only three studies [78,79,81] found no changes in lymphocyte count in response to marathon races in disabled participants. However, other investigations were able to find increased lymphocyte counts in response to low–to-moderate intensity exercises such as arm crank ergometer exercise at 60% of VO_2max_ or electrical stimulation lasting up to 30 min. Similarly, only one study [79] conducted in a sample of Paralympic rowers showed leukocytosis following maximal exercise. Disparities in results may be due to the sample size chosen and the methodology adopted (such as the study design) but, most importantly, to the exercise modality (in terms of duration and intensity). Leukocytosis and then post-exercise lymphopenia constitute the biphasic response to intense exercise [94], even though these “biphasic changes” are generally overlooked and understudied in the disabled athlete population.

As previously mentioned, further explanations of this and other immunological phenomena may be linked to the impact of cortisol and biological amines, such as catecholamines, which are known to be important mediators of immunological responses in a number of different disabilities/impairments, especially in spinal cord injury.

### 4.4. Natural Killer Cells in Disabled Athletes

NK cells are large granular lymphocytes that belong to the innate compartment of the human immune system. They participate in the immunosurveillance of tumors and in the early control of microbial infections. These cells are capable of killing tumor cells while sparing healthy ones according to the “immunological theory of the self”. They also produce proinflammatory cytokines such as interferon-gamma (IFN-y) which participate in the orientation and modulation of the adaptive immune response. NK cells produce other pro-inflammatory cytokines, but also immunoregulatory ones, such as the immunosuppressive IL-10, growth factors, such as the granulocyte-macrophage colony-stimulating factor (GM-CSF) and the granulocyte colony-stimulating factor (G-CSF), as well as other various chemokines.

In disabled people with neurological impairments such as multiple sclerosis, immunopathological studies have largely focused on adaptive T and B lymphocytes. Regarding NK cells, their precise involvement remains unclear and even controversial [95,96]. On the one hand, it has been observed that blocking the infiltration of NK cells into the central nervous system can cause symptom exacerbation, such as in an experimental model of autoimmune encephalitis, but other observations have shown a pro-inflammatory role of the NK cells through their interaction with T and B lymphocytes and antigen-presenting cells. On the other hand, patients suffering from relapsing–remitting multiple sclerosis with significant NK cell activity are more prone to disease progression and more active and developing lesions [95,96].

In other conditions, such as exercise/sport and physical activity, it has been well determined that in able-bodied athletes, regardless of the exercise modality (in terms of duration and intensity), NK activity can increase due to the increase in the number of NK cells. However, according to some observations, NKCA can also stay unchanged or even be reduced depending on the intensity and duration of the exercise [97]. During the exercise, recruited NKs can have a high response to IL-2 stimulation [98]. After a long, intense exercise, the concentration of circulating NK cells and functional NK activity decrease below baseline values. This decrease is maximal 2 to 4 h after exercise [99].

In the present review focused on disabled athletes, disparities in results were noted in the studies overviewed. While some investigations reported decreased NK cells in wheelchair athletes and spinal-cord-injured ones, others reported an increased count or no changes. These discrepancies could be due to the different test/competition chosen for the assessment of exercise-induced changes in NK cells. For instance, elevated NK cell counts were detected in response to long-duration and intense competitions, while decreases or no changes in NK cell levels were detected in response to exercises conducted at lower intensities. Conflicting findings may also be due to compensatory mechanisms specific to the type of disability/impairment under study and involve, for example, other mediators, such as biological amines (catecholamines).

### 4.5. Cytokines and Chemokines in Disabled Athletes

A growing body of scholarly research has consistently shown that exercise may help reduce pro-inflammatory cytokines in a number of circumstances and clinical populations, including former cancer patients [100]. The previously published data suggest that combined aerobic and resistance training may help reduce pro-inflammatory markers in former prostate and breast cancer patients by increasing the counts of lymphocytes, including NK cells [101,102,103,104].

Under normal conditions, immune cells generally do not produce cytokines: they can produce them only after being activated, in general by pathogenic agents or in particular circumstances, including exercise/physical activity. In response to physical exercise, there is an increase in plasma levels of IL-1 and especially IL-6 [105]. After a marathon race, there is also an increase in TNF-α, IL-1β, and IL-6. This elevation is followed by an increase in IL-1RA, soluble TNF receptors, and IL-10 with anti-inflammatory properties [106,107]. Chemokines such as IL-8, MIP1α, and MIP1β can also rise in concentration after a marathon [108]. In the urine, the presence of proinflammatory cytokines was observed after exercise (including TNF-α, IL1β, IL-6, IL-2 receptor, and IFN-γ, among others) [109,110].

Studies overviewed in the present review suggested that cytokines play a major role in the etiopathogenesis of the damage in spinal-cord-injured people as well as during the recovery and rehabilitation periods. Studying the variation of immunological parameters at rest and in response to physical activity/exercise can help find potentially new strategies targeting a specific type of disability/impairment.

Increased IL-6 levels after exercise can be found in wheelchair athletes in some studies, while disparities could be noted in the reporting of TNF-α and hsCRP concentrations, which were the most measured immunological parameters. The discordant results in the studies overviewed in the present review can be explained by taking into account several factors such as the type of physical activity (intensity and duration) and the nature and sensitivity of the test, as well as the precise type of disability/impairment (for instance, level and completeness of spinal cord injury).

### 4.6. Behavioral, Dietary, and Training Strategies in Disabled Athletes

For a person with a disability, participating in sports activities and/or competitions can be a challenging condition for the immune system. The relationship between exercise and immunity response in disabled athletes is, indeed, extremely complex for several reasons, including (1) the chronic low-grade inflammatory and immunodepression—“secondary immune deficiency”—state imposed by the disability/impairment; (2) the impact of the disability on an array of variables, spanning from physical fitness to well-being, quality of life, sleep, and nutritional aspects, among others, which are known to mediate/modulate the effects of exercise on human health; (3) the variability of the parameters related to the exercise/physical activity (modality, frequency, intensity, duration, training versus competition, etc.); and (4) the intra- and inter-individual variability of the immunological response to exercise.

In able-bodied athletes, the previously published data describe several exercise-induced changes affecting various immunological subsets and subpopulations, ranging from neutrophils to lymphocytes, and monocytes. Broadly, moderate-intensity workout is accompanied by optimal immunity and resistance to infections such as URTI in athletes. Periods of intense training with insufficient recovery can cause a temporary state of immunosuppression, which should end with a few days of rest/recovery from exercise. Disabled athletes are relatively overlooked and understudied compared to their able-bodied counterparts. Findings from the few studies available on paralympic and disabled athletes have been here summarized and analyzed utilizing a narrative approach to review and determine the major features of the immunological and inflammatory responses to exercise in this specific population. Moreover, only a few studies have reported behavioral, dietary, and training strategies that can be adopted to limit exercise-induced immunosuppression and reduce the risk of infection in people with disabilities. However, given the paucity of data and contrasting findings, future high-quality investigations on paralympic and disabled athletes are urgently needed.

## 5. Conclusions

In general, a few studies have explored immunological responses in disabled athletes, especially from a comparative perspective (athletes with versus without disabilities/impairments), which would be paramount in dissecting the molecular and cellular basis of the adaptation to exercise in this specific, unique population. Disabled athletes are generally overlooked and understudied, whilst research could help unlock new viable approaches and strategies to counteract the poor health-related outcomes imposed by the disability/impairment.

There is a limited number of studies on the immunological response to physical activity/exercise in people with disabilities on selected immunological parameters. Findings from these few studies suggested no impact of acute and chronic exercise (in terms of training load, intensity, and duration) on salivary antibodies in spinal-cord-injured (tetraplegic and paraplegic) athletes, regardless of the precise type of impairment. However, when it comes to more intense exercises, there was an impaired sIgA secretion rate and α-amylase activity. Only very few studies reported no changes in lymphocyte counts in response to intense exercise (marathon competition), while others suggested an increased count in response to progressive maximal exercise. Similarly, studies reported an increase in lymphocytes when measured in low–moderate intensity exercise in spinal-cord-injured athletes (either in populations of paraplegics and/or tetraplegics). In other words, moderate physical activity seems, therefore, to have a protective effect on the human body in disabled subjects by promoting the detachment of leukocytes and their diffusion in the blood system and by the recruitment of neutrophil cells which can have a “booster effect” on the immune system. Conversely, if the activity is too intense, the natural defense functions will be upgraded to properly adjust the pro- and anti-inflammatory balance. Harnessing these anti-inflammatory abilities may hold the key to new strategies for the treatment of inflammatory diseases, especially in the disabled population, which exhibits a chronic low-grade inflammatory state combined with immunodepression (“secondary immune deficiency”). Furthermore, NK cell count was found unchanged or reduced in spinal cord injury and wheelchair athletes during low intense competition, while it increased in more intensive sporting events in disabled athletes, even though this could be due to the type of disability/impairment (level and completeness of the lesion). Concerning cytokines, increased IL-6 levels were generally reported in wheelchair athletes, while contradictory findings regarding interferons and other chemokines were reported.

Further high-quality investigations are needed to clarify the reasons underlying conflicting findings regarding immunological parameters in disabled athletes, also leveraging molecular big data [106], next-generation immunological assays (i.e., the so-called “immunomics”) [111], and including the measurement of other molecules, such as biological amines (catecholamines) and hormones (cortisol) which are known to mediate/modulate the immunological responses to exercise within a more comprehensive, integrative, and systems-based approach.

## Figures and Tables

**Table 1:** Search strategy adopted in the present review.

Search Strategy	Selection Criteria
Keywords	(interleukin OR cytokine OR chemokine OR myokine OR inflammation OR inflammatory OR immunity OR “immune system” OR “immune response” OR immunological OR monocytes OR lymphocytes OR neutrophils OR phagocytosis OR granulocytes OR “natural killer” OR “NK cells”) AND (“disabled athletes” OR “athletes with disabilities” OR “Paralympics” OR “para-athletes” OR “wheelchair basketball” OR “wheelchair fencing” OR “wheelchair rugby” OR “wheelchair tennis” OR “sitting volleyball” OR ((“Down syndrome” OR “intellectual disability” OR “spinal cord injury” OR paraplegia OR paraplegic OR diplegia OR diplegic OR tetraplegia OR tetraplegic OR quadriplegia OR quadriplegic OR ataxia OR athetosis OR hypertonia OR “motor disability” OR wheelchair) AND (exercise OR “physical activity”))) NOT (rats OR rodents OR mice OR mouse OR “animal model”)
Inclusion criteria	All ages, any sex/gender groups, any para-sports disciplines All paralympic categories, athletes with disabilities, sufficiently trained people with disabilities
Exclusion criteria	Able-bodied athletes Non-paralympic categories/athletes without disabilities People with disabilities undergoing rehabilitation protocols
Hand-searched target journals	Arch Phys Med Rehabil; Br J Sports Med; Endocr Metab Immune Disord Drug Targets; Int J Sport Nutr Exerc Metab; Int J Sports Physiol Perform; J Intellect Disabil Res; Spinal Cord

**Table 2:** Main features of selected studies included in the present systematic review.

Study	Athlete Population	Training/Exercise	Studied Parameters	Major Findings
**Mucosal Humoral Immunity**				
Stephenson et al. [35]	Seven elite para triathletes	34-week training	Weekly training duration, sIgA, URI **Measurements**: Weekly saliva samples	- Negative correlation between weekly training duration and sIgA secretion rate (*p* = 0.028) - No relationships between external or internal training load or URI and sIgA parameters - No significant difference in SIgA when URI is present or not
Leicht et al. [36]	Fourteen elite tetraplegic athletes	Five months of training	Training load, sIgA, URS **Measurements**: Throughout the study’s duration Before 11 am and before any physical activity Self-reported	- Negative correlation between training load and sIgA (*p* = 0.04) - No relationship between sIgA and URS - No difference in SIgA between athletes with and without URS
Leicht et al. [37]	Seven highly trained wheelchair rugby athletes with tetraplegia	Two separate highly strenuous sessions, lasting 23 and 41.5 min	sIgA secretion rate and concentration, and α-amylase **Measurements**: Saliva was collected before, after, and 30 min after exercise Blood lactate before and immediately after the training session	- sIgA secretion rate and α-amylase unaffected by exercise - Increased sIgA concentration post aerobic session accompanied by decreases in saliva flow rate - No difference for saliva in osmolality
Leicht et al. [38]	Twenty-three wheelchair athletes (eight tetraplegics, seven paraplegics, and eight non-spinal cord-injured participants)	Two randomized and counterbalanced 60 min sessions on a treadmill	sIgA secretion rate and α-amylase activity **Measurements**: Before, during, after, and 30 min after exercise	- Increased sIgA secretion rate and α-amylase activity
**Cytokines and Chemokines**				
Kinoshita et al. [60]	Five wheelchair basketball players with spinal cord injuries	2009 Mei-shin League of Wheelchair Basketball Games	Plasma IL-6, TNF-α, CRP levels, blood cell count, and accumulated play duration **Measurements**: 1 h before warm-up and after the game	- Increased plasma IL-6 level and the number of monocytes - Significant relationship between increased IL-6 levels and accumulated play duration - No significant correlation between increased monocyte number and IL-6 level changes
Ogawa et al. [61]	Six athletes with cervical spinal cord injuries and eight athletes with thoracic and lumbar spinal cord injury	30th Oita International Wheelchair Marathon Race	- IL-6 - (TNF)-α - adrenaline and blood cell **Measurements**: Before, immediately after, and 2 h after the race 24 h recovery	- Stable increased monocyte counts (depending on the disability) - Increased IL-6 - Decreased TNF-α
Sasaki et al. [62]	Twenty-eight men with spinal cord injury (16 full-marathon racers, full-group and 12 half-marathon racers, half-group)	28th Oita International Wheelchair Marathon Race, Japan	- IL-6 - (TNF)-α - Sensitivity C-reactive protein **Measurements**: 24 h and immediately before and after the race	- Increased IL-6 - Correlation between plasma IL-6 and hsCRP and average wheelchair speed - TNF-α and hsCRP did not change - No correlation between IL-6 and wheelchair performance
Hoekstra et al. [63]	Seventeen men athletes with cervical spinal cord injury or without	Oita 2016 Wheelchair half-marathon	- Adrenaline and noradrenaline - IL-6 - eHsp72 **Measurements**: Before, after, and 1 h after race	- Large increase in IL-6 after race in NON-CSCI - No change in eHsp72 concentration - Increased plasma adrenaline and noradrenaline levels in NON-CSCI (*p* < 0.036)
An et al. [87]	Ten wheelchair basketball players with spinal cord injuries and amputation (nine in the final test)	Korean Paralympic wheelchair basketball league	Inflammatory response Muscular fatigue **Measurements**: Before the intense exercise and 4 days after recovery	- Significant inhibition of IL-6 levels in the ELAA-treated group - No changes in TNF-a and creatinine kinase levels - Significant improvement in the whole body and back muscle soreness values
**Neutrophils**				
Levada-Pires et al. [73]	Ten male wheelchair basketballers with a spinal cord injury (T1-L3)	Basketball match	Neutrophil levels and function	- Decreased phagocytosis capacity - Increased percentage of cells with the integral plasma membrane - Increased proportion of cells with mitochondrial membrane polarization - Increased neutral lipid accumulation - Increased ROS production
Marques et al. [86]	Eight male wheelchair basketball players	DHA-rich FO supplementation	Neutrophil function	- Loss of membrane integrity - ROS production - High mitochondrial membrane potential - Decreased phagocytic capacity and IL-6 production
**Natural Killer Cells**				
Furusawa et al. [75]	Competitive wheelchair racers with T5-L1 spinal cord injury	Wheelchair full marathon	Peripheral NK cell counts and NKCA	Decreased peripheral NK cells and NKCA
Furusawa et al. [76]	Disabled recreational athletes with T7-L1 spinal cord	Wheelchair half marathon	NKCA	Short-term increase in NKCA
Ueta et al. [78]	Seven spinal-cord-injured individuals	2 h arm crank ergometer exercise at 60% of VO_2max_	NKCA	Decreased NKCA, recovering after 2 h
Yamanaka et al. [82]	Eight spinal-cord-injured individuals (C6-C7)	20-min arm crank ergometer exercise at 60% of VO_2max_	NKCA	No changes in NKCA
Nowak et al. [79]	Paralympic rowers	Paralympic Games in Rio, 2016	NK cell counts	Increased NK cell counts
**Lymphocytes**				
Banno et al. [77]	Six cervical spinal cord injury individuals and seven individuals with T4 and L1 spinal cord injuries in a wheelchair half-marathon race	The 29th Oita International Wheelchair Marathon Race	NKCA cell count **Measurements**: Before, after, and 2 h after recovery	No changes in the lymphocyte counts
Furusawa et al. [75]	Nine male wheelchair marathon athletes with spinal cord injuries between T5 and T12	15th Oita International Wheelchair Marathon Race	NK cell counts **Measurements**: The day before, immediately after, and 1 day after the race	No changes in the lymphocyte counts
Furusawa et al. [76]	Seven men wheelchair racers with spinal cord injuries between T7 and L1	International Wheelchair Marathon Race in Japan	NK cell counts **Measurements**: The day before, immediately after, and 1 day after the race	No changes in the lymphocyte counts
Nash [80]	Individuals with quadriplegia	Cycling exercise	Immune system NK cell counts Cytotoxicity **Measurements**: NA	- No production of an archetypical leukocytosis - Raising in NK cell number and cytotoxicity for 1.5 h after exercise - Immune system is responsive to exercise challenge
Ueta et al. [78]	Seven individuals with spinal cord injury between T11 and L4 Six able-bodied individuals	Arm crank ergometer exercise at 60% of VO_2max_	NKCA **Measurements**: Before, during, and immediately after the exercise	Increased lymphocyte counts (*p* < 0.05) Decreased NKCA in SCI Significant increases in plasma adrenaline No changes in plasma cortisol
Klokker et al. [81]	Eleven subjects with spinal cord injury (five paraplegic individuals and six quadriplegic individuals)	30 min electrically stimulated cycling exercise	NK cells **Measurements**: Before, at the end of the exercise, and 2 h after the exercise	Increased lymphocyte counts
Nowak et al. [79]	Two Paralympic rowers	Rowing ergometer until exhaustion	Cardiorespiratory fitness Lymphocyte counts **Measurements**: Blood samples 3 times The day before testing, 5 min after exercise, and after the recovery period	Increased lymphocyte counts

## Data Availability

Not applicable.
